# Brain Cell Type-Specific Nuclear Proteomics Is Imperative to Resolve Neurodegenerative Disease Mechanisms

**DOI:** 10.3389/fnins.2022.902146

**Published:** 2022-06-16

**Authors:** Ruth S. Nelson, Eric B. Dammer, Juliet V. Santiago, Nicholas T. Seyfried, Srikant Rangaraju

**Affiliations:** ^1^Department of Neurology, Emory University, Atlanta, GA, United States; ^2^Department of Biochemistry, Emory University, Atlanta, GA, United States

**Keywords:** proteomics, nucleus, neurodegeneration, flow cytometry, Alzheimer's disease

## Abstract

Neurodegenerative diseases (NDs) involve complex cellular mechanisms that are incompletely understood. Emerging findings have revealed that disruption of nuclear processes play key roles in ND pathogenesis. The nucleus is a nexus for gene regulation and cellular processes that together, may underlie pathomechanisms of NDs. Furthermore, many genetic risk factors for NDs encode proteins that are either present in the nucleus or are involved in nuclear processes (for example, RNA binding proteins, epigenetic regulators, or nuclear-cytoplasmic transport proteins). While recent advances in nuclear transcriptomics have been significant, studies of the nuclear proteome in brain have been relatively limited. We propose that a comprehensive analysis of nuclear proteomic alterations of various brain cell types in NDs may provide novel biological and therapeutic insights. This may be feasible because emerging technical advances allow isolation and investigation of intact nuclei from post-mortem frozen human brain tissue with cell type-specific and single-cell resolution. Accordingly, nuclei of various brain cell types harbor unique protein markers which can be used to isolate cell-type specific nuclei followed by down-stream proteomics by mass spectrometry. Here we review the literature providing a rationale for investigating proteomic changes occurring in nuclei in NDs and then highlight the potential for brain cell type-specific nuclear proteomics to enhance our understanding of distinct cellular mechanisms that drive ND pathogenesis.

## Introduction

Neurodegenerative diseases (NDs), including Alzheimer's Disease (AD), afflict more than 50 million people worldwide, resulting in lower quality of life for both the afflicted individuals and their caretakers (Prince et al., 2013). With increasing lifespans, the number of individuals living with NDs is projected to double by 2050 (Prince et al., 2016). Effective disease-modifying therapies for most NDs are currently lacking although some progress has been made recently in AD therapeutics (Briggs et al., 2016; Sevigny et al., 2016; Tanzi, 2021).

Different cell types of the central nervous system (CNS) including neurons, microglia, astrocytes, oligodendrocytes and endothelial cells are affected by (and may in turn contribute to) ND pathology in a cell-type specific manner. To highlight this, De Strooper and Karran describe a “cellular phase” of AD, which follows a “biochemical phase” characterized by amyloid beta accumulation and tau hyperphosphorylation (Strooper et al., 2016), in which each cell-type has a unique phenotype invested with a profile of mechanisms which react and contribute to disease pathogenesis (Strooper et al., 2016). These cell-type specific mechanisms can either accelerate or hamper disease pathogenesis. Thus, resolving mechanisms of NDs requires molecular characterization of distinct CNS cell types.

While bulk and single nuclear transcriptomics of post-mortem human brain have provided several novel insights into cell type-specific ND mechanisms, transcriptomic data is poorly reflective of functional protein-level abundance data (Seyfried et al., 2017; Higginbotham et al., 2020). This data may be captured by unbiased proteomics strategies such as mass spectrometry. Unfortunately, proteomics of different CNS cell types from human brain is limited due to limited fresh brain tissue availability and low recovery of CNS cell types for downstream proteomic analyses. Even from fresh human brain, recovery of intact astrocytes, oligodendrocytes and neurons is highly limited (Kelley et al., 2018). However, unlike the cell membrane, the nucleus of the cell retains its structural integrity even in frozen brain specimens. This feature allows for the potential to isolate cell-type specific nuclei from archived brain specimens. Proteomics of purified nuclei from the brain, therefore, holds significant potential to elucidate disease-driving mechanisms in humans.

The nucleus is a membrane-bound organelle within eukaryotic cells that houses the genome, regulates cellular activities, and plays critical roles in cellular homeostasis and disease (Guo and Fang, 2014). Proteins that reside in the nucleus or traffic to the nucleus regulate chromatin structure and folding, DNA replication, RNA synthesis and splicing, and orchestrate gene expression programs (Dundr and Misteli, 2001). Therefore, characterization of the proteomic composition of nuclei could provide a wealth of information regarding disease processes. This could be done by leveraging existing biorepositories of non-fixed frozen human brain tissues across several NDs.

In this review, we highlight nuclear processes that play pathophysiological roles in NDs and discuss how CNS cell-type specific nuclear proteomics can provide critical insight into disease pathogenesis. Last, we review emerging methodologies for cell type-specific nuclear proteomics using human brain tissues.

## Structure and Sub-Compartments of the Nucleus

**Figure 1:**
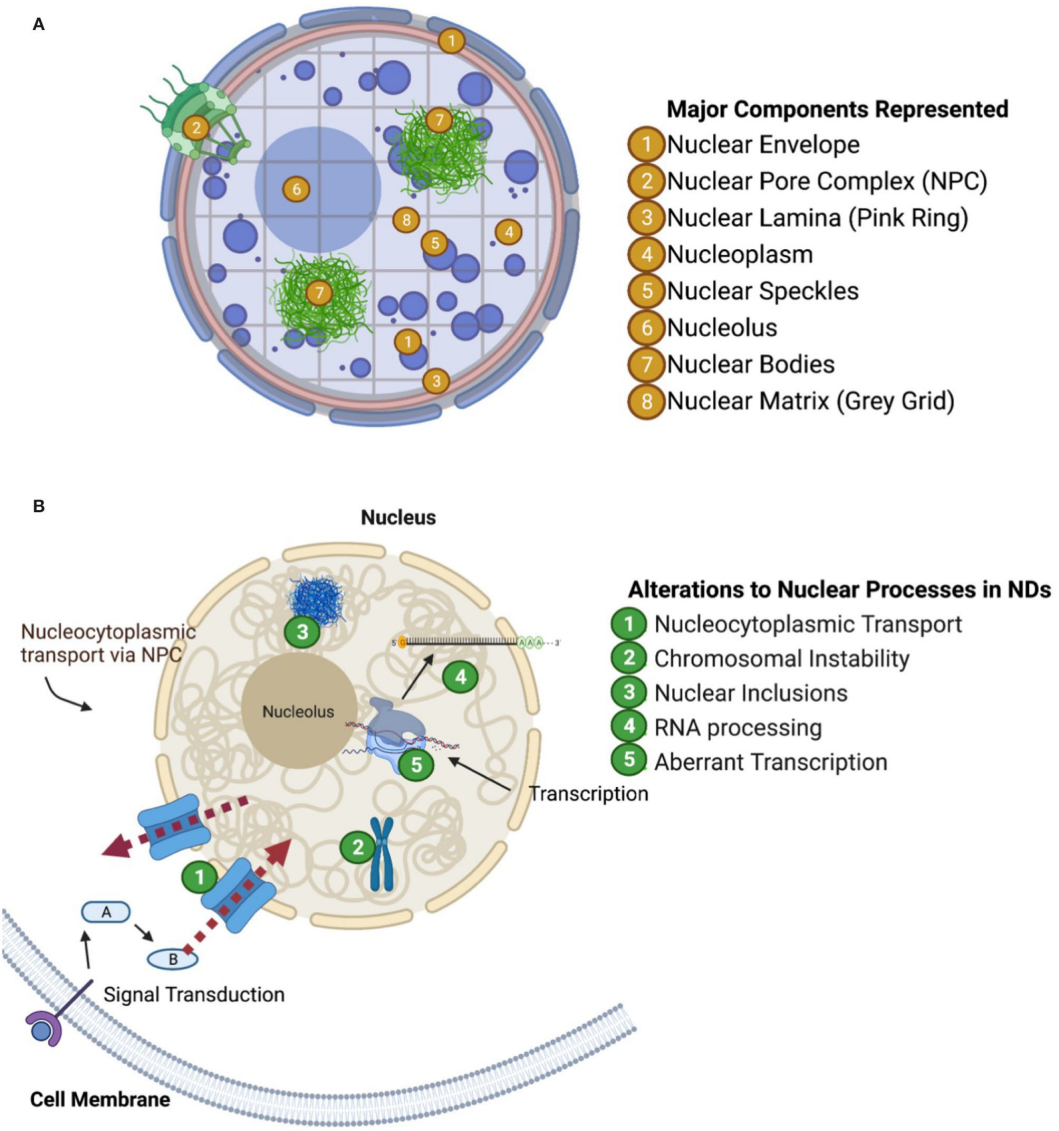
Nuclear compartments and functional groups of proteins perturbed in neurodegenerative diseases. **(A)** Visualization of nuclear structures (one way to characterize nuclear proteins). **(B)** Visualization of aberrant nuclear mechanisms in neurodegeneration as discussed in paper.

Proteins contained within the nucleus can be characterized by their functional role in the nucleus and by their compartmental localization in the nucleus. Unlike a cell which is organized into subcellular organelles enclosed by membranes, the cell nucleus is “spatially organized” into territories. The nucleus is composed of distinct spatially organized compartments, such as the nuclear envelope, nucleolus, nuclear speckles, nuclear bodies, chromatin, and the nuclear lamina and matrix which are represented in Figure 1A. Generally, distinct chromosomes fold themselves into separate territories of chromatin, DNA coiled around nucleosomes made of histone proteins. Chromatin tends to arrange into configurations which co-localize genes with similar functions (including those on different chromosomes). For example, genes encoding ribosome subunits, rDNA, localized into Nucleolar Organizing Regions (NORs) which compose the nucleolus (Thompson et al., 2003; Kobayashi, 2008; Meldi and Brickner, 2011). The nucleolus is a complex structure composed of DNA, RNA, and proteins which regulate ribosome biogenesis.

The nucleus also houses structures which provide structural and mechanical support, such as the nuclear lamina and the nuclear matrix. The nuclear lamina is associated with the inner membrane of the nuclear envelope and is composed of various lipids and membrane proteins. The nuclear lamina helps retain the structure of the nucleus, organize chromatin and chromatin folding, and anchor the nuclear pore complex in the nuclear envelope. The nuclear matrix is similar in function to the nuclear lamina in that it provides structural support, but it extends throughout the nucleoplasm providing a scaffold upon which chromosomes can fold and organize themselves. Both of these structures associate to specific regions of repressed heterochromatin called Lamina Associated Domains and Matrix Associated Domains, respectively (Guerreiro and Kind, 2019). The association of these chromatin domains to their respective structure aids in folding of chromosomes as well as the maintenance of generally transcriptionally repressed heterochromatin. In this way, these components not only maintain the structural organization of the nucleus, but also reinforce cell-type specific regulation of gene expression.

Additionally, proteins and RNA can localize into distinct structures called “nuclear bodies” which can regulate key biological processes such as transcription, mRNA splicing and DNA repair. There are several well-known nuclear body subtypes such as the Histone Locus Body, the Cajal Body, and the Promyelocytic Leukemia nuclear body. During transcriptional regulation, it is proposed that genes could either move to nuclear bodies or that the appropriate nuclear bodies could form *de novo* at transcriptional sites or at specific chromosomal loci.

Finally, the nuclear speckles represent another group of nuclear domains which are enriched with pre-mRNA splicing factors such as small nuclear ribonucleoproteins (snRNPs) and Serine/Arginine-rich (SR) proteins (Fu, 1995). Many other regulatory factors have been found in the nuclear speckles which suggest their multi-functional roles in nuclear homeostasis. Nuclear speckles are dynamic structures, where some constituents with low complexity (LC) or LC prion-like domains can interact and reversibly self-aggregate (Galganski et al., 2017). However, unlike SR proteins and heterogeneous nuclear ribonucleoproteins (hnRNPs) which have prion-like domains that can bind RNA to dynamically enhance or suppress splicing sites, snRNPs are more structurally rigid protein complexes scaffolded by snRNA (Chatel and Fahrenkrog, 2012; Bai et al., 2013; Diner et al., 2014; Xue et al., 2019; Khan et al., 2020).

In conclusion, the nucleus has functionally distinct compartments which are spatially organized rather than organized by membranes (Figure 1A). These distinct compartments are vital to the homeostatic maintenance of DNA transcription, and mRNA production, maturation, and export among other nuclear functions. Proteins' localization to specific nuclear compartments helps determine their functional role. As we examine nuclear proteomes during ND pathogenesis, we may additionally consider pairing observations on the subnuclear localization of significantly altered proteins, e.g., among associated chromatin domains, as well as determining how major nuclear components are altered longitudinally over the course of ND pathogenesis.

## Pathophysiological Roles of the Nucleus in Neurodegenerative Diseases

The relevance of the nucleus in NDs is indicated by the results of genomic studies, which have found that nuclear-expressed gene products are risk factors for disease. Findings from genome-wide association studies (GWAS) have helped define the genetic risk architecture underlying NDs including late onset AD (LOAD) (Lambert et al., 2013), amyotrophic lateral sclerosis (ALS) (van Rheenen et al., 2016), frontotemporal dementia (FTD) (Ferrari et al., 2014), Huntington's disease (HD) [Genetic Modifiers of Huntington's Disease (GeM-HD) Consortium, 2015; Moss et al., 2017], and Parkinson's Disease (PD) (Grenn et al., 2020). Nuclear expression and/or the impact on nuclear biology are common themes among risk-associated genes across these NDs (Leeuw et al., 2015; Kunkle et al., 2019). A high-level summary of nuclear mechanisms that are disrupted in NDs is provided in Figure 1B.

One critical paradigm related to the genetic determinants of NDs are genomic repeat expansions. These genetic variants can have strong impacts on nuclear biology and are causally linked to several known NDs. For example, expansions of “triplet” CAG repeats within protein coding regions cause Spinal Bulbar Muscular Atrophy (SBMA), Huntington's Disease (HD), Spinocerebellar Ataxia type 1 (SCA1), and Dentatorubral-Pallidoluysian Atrophy (DRPLA) (La Spada et al., 1994). In addition, GGGGCC hexanucleotide repeat expansions in non-protein coding regions of the *C9ORF72* gene can cause ALS and FTD (DeJesus-Hernandez et al., 2011). While the mechanism by which these mutations contribute to disease pathogenesis is controversial in some diseases, it is suspected that these mutations cause aberrant nucleocytoplasmic transport as shown by the pathological sequestering of RNA binding proteins in the nucleus (Zhang et al., 2015). Repeat-associated non-AUG translation is another mechanism of disease pathogenesis that occurs due to initiation of translation at disease-causing repeat expansions, leading to toxic protein accumulation (Green et al., 2017). Notably, the translated protein products of these repeat expansions encode LC peptides, some of which undergo liquid-liquid phase separation. This phase separation can affect membraneless organelle (all sub-compartments of the nucleus) formation and thus nuclear processes including transcription (Chen et al., 2021).

**Figure 2:**
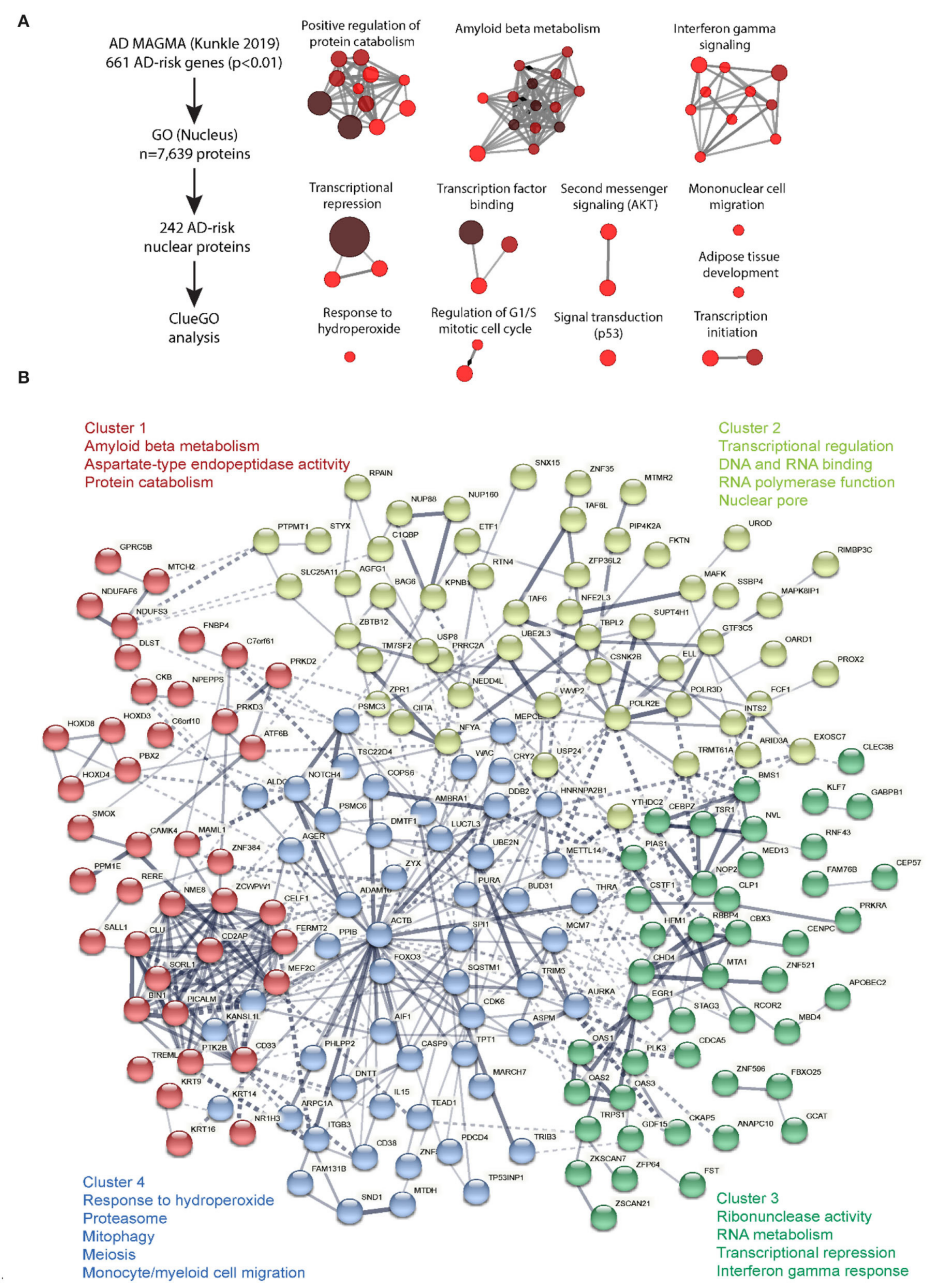
ClueGO analysis of cell-type specific proteome. Molecular mechanisms regulated by nuclear-expressed proteins of AD relevance. **(A)** ClueGO analysis was performed on the 242 proteins encoded by AD risk genes as identified by MAGMA analysis of human AD GWAS (Kunkle et al., 2019), and with known nuclear function (based on GO Nucleus term membership). This analysis identified 12 clusters of biological and molecular processes. Each node represents a GO term and connectivity indicates shared gene symbols. Size of the node represents strength of statistical significance and intensity of color represents number of genes (fewer: red, higher: brown). **(B)** STRING analysis was performed on these 242 AD risk nuclear proteins to identify functional groups of proteins based on protein-protein interactions (PPI), either functional and/or physical interactions. K means clustering revealed 4 clusters of proteins and key GO terms for each cluster have been shown. Conntectivity represents known PPI (thickness represents strength of connectivity between two proteins). Gene symbols for each are indicated.

Analysis of genetic risk markers have implicated nuclear biology in the pathogenesis of many other NDs including LOAD. Multi-marker analysis of genomic annotation (MAGMA) of GWAS studies of over 100,000 subjects have used SNP association data to nominate 1,822 genes with potential causal links to LOAD (Jansen et al., 2019; Kunkle et al., 2019; Johnson et al., 2020). To determine whether these LOAD risk genes include an overrepresentation of genes that encode proteins with known nucleus-associated localization or function, we obtained a list of 7,639 protein-coding gene symbols that were assigned to the Gene Ontology (GO) term “nucleus” (GO term 0005634). Of the 1,822 MAGMA-identified LOAD risk genes, 682 were annotated as producing at least one nuclear localized isoform, representing approximately a third of LOAD risk genes. From this list, we filtered 242 genes with MAGMA significance of *p*-value < 0.01) with assignment to “nucleus” GO category and performed pathway analyses (ClueGO v2.5.8, a plugin within Cytoscape v3.5) (Chatel and Fahrenkrog, 2012). Pathway analysis of this intersection of nucleus-related genes pertaining to LOAD risk identified groups of genes (Figure 2, Supplementary Data 1) involved in regulation of protein catabolism, amyloid beta metabolism, response to redox stress, repression of gene transcription, interferon signaling, and signaling pathways. Specific genes related to RNA polymerase I expression, ribosomal RNA expression and gene expression included *CBX3, CHD4, ERCC2, MTA1, POLR2E, ACTB*, and those involved in nucleotide excision repair included *ELL, ERCC1, ERCC2, POLR2E, UBE2N*. While this GO term-based analysis of LOAD risk genes does not imply exclusive nuclear localization or nucleus-associated function of these proteins, it highlights the potential importance of nuclear biology in LOAD pathogenesis.

In addition to genetic insights from GWAS and repeat expansion disorders, a number of different nuclei-related mechanisms are implicated in NDs. Here we highlight a subset of these mechanisms (Figure 1B) including nucleocytoplasmic transport (NCT), chromosomal maintenance and stability, RNA processing *via* protein association, and specific proteins misfolding into “inclusion body”-type pathology within the nucleus. Examining the abundance, post-translational modifications and localization of nuclear proteins in neurodegeneration could provide valuable information about ND-driving mechanisms. Below, we briefly review these distinct mechanisms in the context of NDs. To accompany the discussion below, Table 1 displays specific proteins and NDs which are associated with each of the mechanisms discussed.

**Table 1:** Specific neurodegenerative diseases and aberrant proteins associated with each mechanism.

**Aberrant nuclear mechanism**	**Neurodegenerative disease**	**Aberrant protein**
Nucleocytoplasmic transport	ALS/FTLD	FUS, TDP-43
	AD	Tau
Chromosomal instability	AD	CDT2, Tau
Nuclear inclusions	NIID	SUMO-1
RNA processing	ALS/FTD	ATXN2, Optineurin, Angiogen
	Myotonic Dystrophy	DMPK, MAPT
	Tauopathies	TIA1
	AD	Tau, BIN1, PiCALM, PTK2B, FERMT
Transcription	AD	BACH1, ERG
	ALS/FTD	SOD1, TARDP
	PD	TFEB

### Nucleocytoplasmic Transport (NCT)

One key nuclear process that has been implicated in ND pathogenesis is NCT (Zhang et al., 2015; Boeynaems et al., 2016; Gasset-Rosa et al., 2017; Grima et al., 2017; Eftekharzadeh et al., 2018). ND-related NCT dysfunction can manifest in both the failure to import and retain normally nuclear material and failure to export normally cytoplasm-bound material. The resulting mislocalized macromolecules include both proteins and RNA (DiFiglia et al., 1997; Amador-Ortiz et al., 2007; Bichelmeier et al., 2007; Dormann et al., 2010; Gasset-Rosa et al., 2017; Grima et al., 2017; Chou et al., 2018; Eftekharzadeh et al., 2018). The current knowledge of the mechanisms underlying NCT dysfunction in neurodegeneration is limited. Possible mechanisms include (mis)targeting of the proteins/mRNA themselves, dysfunction of the nuclear transport receptors (NTRs) (Kapinos et al., 2017), or of the nuclear pore complex (NPC) (Ori et al., 2013; Sakiyama et al., 2016; Eustice et al., 2017). Despite the incomplete knowledge to date, there are compelling indications that NCT dysfunction contributes to NDs (Zhang et al., 2015; Boeynaems et al., 2016; Gasset-Rosa et al., 2017; Grima et al., 2017; Eftekharzadeh et al., 2018).

NCT dysfunction has been implicated in FUS and TAR-DNA binding protein 43 kDa (TDP-43) proteinopathies because both of these proteins are normally enriched in the nucleus, although in ALS/FTD spectrum disorders, they are mislocalized in the cytoplasm (Arai et al., 2006; Dormann et al., 2010). This mislocalization of FUS/TDP-43 is conspicuous in FTD-TDP, ALS, and in Limbic-predominant age-related TDP-43 encephalopathy (LATE). Some cases of ALS are caused by mutations in the nuclear localization signal (NLS) of the FUS protein (Belzil et al., 2009; Lopez-Erauskin et al., 2018) which disable the translocation of FUS to the nucleus (Dormann et al., 2012). It has been hypothesized that the mislocalization of FUS and TDP-43 causes a loss-of-function disease pathogenesis by disabling their nuclear functions of transcription and pre-mRNA splicing, and also a gain-of-function toxic impact in the cytosolic compartment (Shelkovnikova et al., 2014; Suk and Rousseaux, 2020).

In AD, tau proteins have been hypothesized to play active roles in NCT function and dysfunction (Eftekharzadeh et al., 2018). Not only are tau proteins apparently mislocalized themselves, but they also interact with nucleoporins of the NPC, amplifying the disruption of NCT (Eftekharzadeh et al., 2018). In addition, NCT dysfunction may also be a downstream effect of pathogenetic germline DNA repeat expansions which are linked to several neurodegenerative diseases. For example, *C9ORF72* hexanucleotide expansion in patients with FTD and ALS is linked to the failure to transport TDP-43 from the cytoplasm to the nucleus (Balendra and Isaacs, 2018; Zhang et al., 2018). Triplet repeat disorders have also been shown to sequester RNA-binding proteins, which can disrupt NCT (Nalavade et al., 2013; Zhang et al., 2018). To further test whether mislocalization of other molecular entities could be attributed to NCT dysfunction, protein transport assays have been developed and performed (Shani et al., 2017; Hutten and Dormann, 2020). These assays can quantitatively measure the nuclear cargoes imported and/or exported using photobleaching assays or import assays *in vitro* or in intact cells (Zhang et al., 2015; Eftekharzadeh et al., 2018; Hutten and Dormann, 2020). The same methodology may in the future be combined with proteomics methods to expand the understanding of NCT in healthy and disease states.

### Chromosomal Proteins

Chromosome-associated proteins provide complex structural and functional support for the genome and gene regulation, and have been shown to be affected in AD and other NDs (Iourov et al., 2009; Arendt et al., 2010; Potter et al., 2019; Yurov et al., 2019). Chromosomal function and stability are crucial to the homeostatic maintenance within the nuclei of all cell types by reducing aneuploidy (abnormal number of chromosomes in a cell), as well as protecting against chromosomal damage, deletions, insertions, or other chromosomal aberrations (Masai et al., 2010; Vijg and Suh, 2013).

Recent research has demonstrated a strong association between chromosomal instability (CIN) and aging-related neuronal deterioration. CIN mediates neuronal loss and acts as a key feature of the pathogenic cascade in NDs (Yurov et al., 2019). This is evidenced by the obstruction of DNA repair and replication efficiency observed in neurodegeneration which is correlated with CIN (Jeppesen et al., 2011; Yurov et al., 2011). Additionally, neurons with chromosomal aberrations or aneuploidy are much more susceptible to cell death, potentially underlying the shared theme of age-related or regional vulnerability of neurons in different NDs (Arendt et al., 2010). Arendt et al. assert that 20–30% of neurons are aneuploid during early stages of AD, and that aneuploid neurons can account for 90% of neuronal loss observed at autopsy (Arendt et al., 2010). Understanding triggers and mechanisms of CIN and/or aneuploidy in neurons and other CNS cell types could provide valuable information for not only the nuclear pathogenesis of neurodegeneration but also possible future drug targets.

### RNA Processing

RNA processing is another crucial aspect of nuclear homeostatic maintenance that may be disrupted in ND pathogenesis (Hsieh et al., 2019). Key players in this process are RNA binding proteins (RBPs) which regulate post-transcriptional gene regulation as well as mRNA splicing, transport, export, and localization (Gerstberger et al., 2014). Therefore, RBPs are partly responsible for the diversity of the proteome. RBP-encoding transcripts as well as genetic mutations in RBPs have been shown to be associated with NDs, including ALS and FTD, where mutations in *TARDP, FUS, ATXN2, MATR3, TIA-1, HNRNPA1*, and *HNRNPA2B1* occur (Sreedharan et al., 2008; Kwiatkowski et al., 2009; Kim et al., 2013a; Johnson et al., 2014; Conlon and Manley, 2017; Kapeli et al., 2017; Mackenzie et al., 2017; Abramzon et al., 2020). In NDs, some of the cognate proteins of these RBP genes become mislocalized (e.g., TDP-43 and FUS), and may be observed in cytosolic stress granules (Arai et al., 2006; Ederle and Dormann, 2017). This mislocalization likely causes loss of normal homeostatic function of these RBPs, resulting in problems in the maturation, splicing, export, and translation of mRNA (Ling et al., 2013). Evidence for this dysregulation may be visible at the proteomic level, displaying incorrectly and dysfunctionally spliced or folded proteins. A proteomic investigation of the nucleus across different cell types and conditions could provide insight into the effect of ND pathogenesis on RBP localization that would not otherwise be revealed by bulk brain proteomics or whole-cell proteomics. Nucleus-specific proteomics could also identify the resulting effect that RBP mislocalization has on protein products that maintain homeostasis in the nucleus.

One disease that is partially attributable to RBPs is myotonic dystrophy (MD) (Lee and Cooper, 2009). MD Type 1 is caused by repeat (CTG)_n_ expansion in the 3′ untranslated region of the *DMPK* gene (Takahashi and Ishiura, 1999; Orengo et al., 2008). When the expansion becomes large enough, the *DMPK* transcript binds and sequesters RBPs in the nuclei of cells throughout the body, including in neurons (Lee and Cooper, 2009). The sequestration of RBPs in MD leads to a deleterious, positively reinforcing cycle of mRNA mis-processing and aberrant splicing. Among the MD Type 1-associated phenomena is mis-splicing of the *MAPT* gene which encodes the protein tau. Tau “tangles” are a characteristic pathologic biomarker of MD type 1, similar to tau neurofibrillary tangles (NFTs) in AD (Caillet-Boudin et al., 2014). MD Type 1 affected individuals develop a tauopathy due to the mis-splicing of tau transcripts in the cell nucleus (Sergeant et al., 2001; Park et al., 2016). Stress granules containing aberrant RBPs were found to be correlated with tau NFT pathology (Maziuk et al., 2018). Additionally, an *in vitro* study by Apicco et al. determined that reducing levels of the RNA binding protein, TIA-1, protects against the accumulation of tau oligomers and increased neuronal survival (Apicco et al., 2018). This provides further indication that RBPs play a role in tau-mediated neurodegeneration.

A relevant bulk brain proteomic analysis of post-mortem human cases, performed by Johnson et al., discerned that RBPs were a class of proteins which were differentially expressed in asymptomatic AD (persons with presumed preclinical disease, or a high pathologic load still below the threshold that generates cognitive impairment), and symptomatic AD (Johnson et al., 2018). Johnson et al. also investigated how loss of splicing function of RNA binding proteins became evident at the protein level by utilizing a proteogenomic approach which examined alternative exon-exon junctions in AD risk proteins such as BIN1, PICALM, PTK2B, and FERMT2 (Johnson et al., 2014). Based on these observations, it is likely that a nucleus-specific proteomic analysis could yield further insights into aberrant splicing machinery mechanisms involving RBPs in NDs.

### Nuclear Inclusion Bodies

Although cytoplasmic inclusion bodies, neuritic (Langfelder and Horvath, 2008) (axonal and dendritic) and extracellular protein accumulation is associated with various NDs, proteinaceous deposits may also occur within cell nuclei. Such deposits may be pathognomonic, as is the case of neuronal Intranuclear Inclusion Disease (NIID) (Sone, 2020). NIID is characterized by eosinophilic hyaline intranuclear inclusions in both the CNS and PNS (Sone et al., 2016). Nuclear inclusions are also seen in other conditions including FTD-TDP, HD, and others (Sieradzan et al., 1999; Woulfe et al., 2001).

NIID is caused by a CGG repeat expansion in the 5′UTR of the *NOTCH2NLC* (a gene that plays roles in neuronal development by regulating Notch signaling), was discovered in Japanese populations and recently replicated in a European ancestry cohort (Nakamura et al., 2020). The NIID intranuclear inclusions are immunoreactive for ubiquitin, p62/SQSTM1, SUMO1, FUS, and OPTN, suggesting that nuclear ubiquitin-mediated proteasome pathways are normally functioning in the nucleus and those pathways are aberrantly stimulated in NIID (Pountney et al., 2003; Franic et al., 2021). Nuclear inclusions are also seen in other conditions including FTLD-TDP, HD, and others (Sieradzan et al., 1999; Woulfe et al., 2001).

### Post-translational Modifications

Post-translational modifications (PTMs) of proteins contribute to the functional diversity of the human proteome by covalently attaching functional groups to amino acids, impacting a protein's localization, functionality, (re)folding and stability. These PTMs include phosphorylation, ubiquitination, and methylation, among many others. Maintenance of homeostatic PTMs is critical to retaining a cell's function and health.

Phosphorylation is one of the most widespread PTMs, and homeostatic regulation of phosphorylation by protein kinases and protein phosphatases can drastically alter the functionality of target proteins. Aberrant hyper-phosphorylation is seen in many NDs, including tauopathies (in which tau becomes hyperphosphorylated and forms NFTs) and TDP-43 proteinopathies (where C-terminal phosphorylated TDP-43 is aberrantly localized outside the nucleus). Hyperphosphorylation of specific amino acid residues on tau is seen to directly incite conformational changes in the protein. Tau hyperphosphorylation is further implicated in neuronal toxicity because hyperphosphorylation of tau precedes NFT aggregation and is also seen to contribute to neuronal toxicity independent of the aggregation of NFTs (Sato-Harada et al., 1996; Cowan et al., 2010; Hoover et al., 2010; Didonna et al., 2019). Phosphorylation is also implicated in other pathologies such as Parkinson's disease, in which aberrant alpha-synuclein aggregates into cytoplasmic inclusions called “Lewy Bodies.” Several phosphorylation sites undergo aberrant phosphorylation in alpha-synuclein proteins, such as Ser129, which is phosphorylated in 4% of homeostatic alpha-synuclein and in 90% of Lewy Body alpha-synuclein (Anderson et al., 2006). The aberrant phosphorylation of Ser129 is seen to modulate the transport of alpha-synuclein between the nucleus and cytoplasm (Goncalves and Outeiro, 2013).

Other PTMs such as aberrant acetylation and ubiquitination are also seen in several NDs. In triplet repeat expansion disorders (such as pathogenetic GAA triplet expansions in Friedreich ataxia or Polyglutamine diseases such as HD) hypo-acetylation of histone proteins can decrease the expression of specific proteins or cause widespread transcriptional deficits at several loci (He and Todd, 2011; Nageshwaran and Festenstein, 2015). Furthermore, ubiquitin is found in pathological protein aggregates in proteinopathies, implicating aberrant regulation of the ubiquitin-proteasome system which is meant to rid a cell of misfolded proteins (Schmidt et al., 2021).

Proteomic studies are able to detect the presence and location of PTMs such as phosphorylation, acetylation, and ubiquitination (Mann and Jensen, 2003). Such modifications can be monitored at different stages of NDs and across different cell types, providing novel proteomic insights in NDs that would otherwise go unnoticed by genomic or transcriptomic approaches.

## The Role of TDP-43 and TAU Proteins in the Nucleus in NDs

### TAR DNA-Binding Protein 43 (TDP-43)

TDP-43 is a nuclear-enriched protein that binds both DNA and RNA (Furukawa et al., 2016) and is expressed throughout many organs and cell types. TDP-43 plays key roles in RNA processing including regulating mRNA splicing and stability (Arnold et al., 2013; Donde et al., 2019). TDP-43 proteinopathy is now known to be a pathologic hallmark in multiple NDs including FTLD, ALS, and LATE (Feneberg et al., 2018; Nelson et al., 2019; Prasad et al., 2019). Under homeostatic conditions, TDP-43 is dephosphorylated at critical residues including serine 403/404 and 409/410 and localized in the nucleus (Hasegawa et al., 2008; Inukai et al., 2008; Mackenzie et al., 2011). However, under pathological conditions and cellular stress, TDP-43 appears in cellular microdomains called stress granules in the cytoplasm (Khalfallah et al., 2018). In dementias, the cytoplasmic TDP-43 can become hyperphosphorylated (including at the aforementioned critical residues) and arrayed in fibrillary polymers (Prasad et al., 2019). Dysfunction of the NPC may contribute to TDP-43 mislocalization and cytoplasmic aggregation in a deleterious feed-forward mechanism (Chou et al., 2018). In this hypothetical framework, TDP-43 aggregates can induce defects in the NPC, leading to dysfunction in NCT *via* mislocalization of nucleoporins and transport factors, which may result in more severe TDP-43 pathology. While TDP-43 proteinopathy was initially discovered in the context of ALS/FTD spectrum disorders, TDP-43 proteinopathy is also a pathological hallmark of the more common ND, Limbic-predominant Age-related TDP-43 Encephalopathy (LATE). While FTLD and ALS afflict ~1/1,000 individuals, LATE afflicts ~1/3 persons above the age of 80 (Hogan et al., 2016; Talbott et al., 2016). The impact of a nuclear-enriched protein such as TDP-43 in a broad spectrum of different diseases—some rare, some very common—underscores the relevance of the nuclear proteome to NDs. Because TDP-43 proteinopathy is an essential marker in many NDs and it is predominantly nuclear, changes of nuclear TDP-43 across cell-types and disease phenotypes could provide valuable cell autonomous and non-cell autonomous insights into the nuclear landscape of ND pathogenesis.

### Tau Protein

Tau protein isoforms result from alternate splicing of the microtubule associated protein (*MAPT*) gene (Andreadis, 2006). These proteins are found in both neuronal cells, and to a lesser degree, non-neuronal cells in the CNS (Guo et al., 2017). Tau proteins are most well-known for their role in microtubule assembly and stability, but they also assist in additional functions such as axonal transport (Guo et al., 2017). Aggregated, hyperphosphorylated tau intracellular inclusions characterize “tauopathies,” which are a subclass of NDs that include AD and FTLD subtypes (Kovacs, 2017).

Tau has been found to be present in the nuclei of human brain cells, where it is predominantly present in nucleoli (Brady et al., 1995; Greenwood and Johnson, 1995; Thurston et al., 1996). Different tau isoforms resulting from alternative splicing, localize to different cellular compartments (Goedert et al., 1989; Xia et al., 2016). Additionally, immunofluorescence and western blot analyses indicate that the majority of tau in the nucleus is dephosphorylated (Arrasate et al., 2000). Ortega et al. determined that nuclear dephosphorylated tau was diminished in the CA1 and dentate gyrus (hippocampal) regions of diseased tissue, and further, practically disappeared in the nuclei of neurons with tau tangles (Hernandez-Ortega et al., 2016). This insight in addition to the known nature of tau-mediated neurodegeneration provides reason to investigate the impact of both homeostatic and diseased tau on nuclear mechanisms. *Via* separate mechanisms, tau may impact both DNA stability and NCT.

Abnormal tau is implicated in CIN by both the effects of tau hyper- phosphorylation (as seen in tauopathies) and by mutations of the *MAPT* gene affecting increased instability and vulnerability of chromatin (Rossi et al., 2013; Alonso et al., 2018; Colnaghi et al., 2020). Lu et al. determined that hyperphosphorylated nuclear tau lost its ability to bind to DNA and thereby to also protect the cell from differing stressors including thermal denaturation and reactive oxygen species attack (Lu et al., 2013). Hua and He also found that nuclear tau protein, both in its native and phosphorylated states, had the ability to bind to and stabilize DNA (Hua and He, 2002). They also determined that aggregated (pathologic) tau lost its ability to bind to and support DNA. These results indicate that as tau proteins become phosphorylated and form aggregates in NDs such as AD, they lose their ability to stabilize DNA.

In addition to investigating the effect of aberrant phosphorylation of tau proteins on CIN, Rossi et al. assessed the effect of different mutations in the *MAPT* gene on the ability of tau to stabilize chromatin, further implicating nuclear tau in chromosomal stability (Rossi et al., 2013; Bukar Maina et al., 2016). Furthermore, tau motifs that bind to DNA can also become phosphorylated in neurodegeneration (Guo et al., 2017; Kimura et al., 2018). It was hypothesized that tau protects DNA by binding to the DNA backbone, which induces an adaptive conformational change, this mechanism has been seen in other DNA protecting proteins (Multhaup et al., 2015; Guo et al., 2017).

In addition to chromosomal stability, tau is implicated in nuclear processes by its effect on NCT. A recent study by Eftekharzadeh et al. revealed that cytosolic tau interacts with the NPCs and that cytosolic tau impacts NCT function (Eftekharzadeh et al., 2018; Diez and Wegmann, 2020). The conditions under which tau is localized in the nucleus, how this effects CIN, and how nuclear tau effects NCT are still controversial topics that warrant further investigation. Proteomic studies focusing on how tau is affected by and correlated with proteins that regulate different nuclear mechanisms could help develop our understanding of these mechanisms.

## Network Based Proteomics in Neurodegeneration

The development of network-based proteomics in recent years has contributed considerably to our understanding of ND pathogenesis (Seyfried et al., 2017; Johnson et al., 2020, 2022; Rayaprolu et al., 2021). Proteomics using mass spectrometry-based approaches enables high-throughput analyses of protein expression using unbiased and targeted approaches. Network-based analyses of proteomic data can help elucidate how the proteins interact with and affect each other by organizing large-scale proteomic data into “modules” of co-expressed proteins (Langfelder and Horvath, 2008). The proteins in these modules are likely to share biological characteristics, cellular functions and potentially, upstream regulation. Individual modules can then be analyzed for their associations with markers of specific cell types, organelles, biological factors, genetic risk factors and disease traits (Rayaprolu et al., 2021). By associating proteomic network modules to quantitative measures of disease pathogenesis, these studies provide insight into the mechanistic factors that are altered in the course of disease pathogenesis and provide a framework for validation and mechanistic studies. This approach, summarized by Rayaprolu et al., highlights six primary modules associated with AD, three of which are increased in AD, and three of which are decreased in AD. These modules are characterized by their cell-type specificity, biological pathways and ontologies, and “common hubs” seen across networks of ND brain proteomes (Rayaprolu et al., 2021).

One strong indicator of the value of network-based brain proteomics approaches in ND research is by its comparison to transcriptomic studies. Modules in the network transcriptome and the network proteome only overlap by 30–40% (Seyfried et al., 2017; Higginbotham et al., 2020). This suggests that there are significant post-transcriptional and post-translational effects occurring in the AD brain in addition to genetic and transcriptional alterations. Thus, it is evident that proteomic studies provide crucial information regarding disease pathogenesis that cannot be obtained from transcriptomic studies.

Network based proteomics of human post-mortem brain samples have mostly been “bulk” tissue analyses, meaning that proteomic analyses were performed on the entirety of the tissue. While bulk proteomic analyses of diseased and control brain tissue have provided valuable insight into alterations at a global scale, we hypothesize that more nuanced proteomic investigations will provide complementary information. By solely examining the proteome at a global level, significant disease related alterations may be missed. These alterations may be missed because they are not deemed significant on the global level, or because they may localize differently in neurodegeneration, but retain similar global abundance levels. For example, a given protein may be homeostatic in astrocytes and disease-driving in neurons, or differentially distributed in cytoplasm and nuclei. Furthermore, alterations in relatively rare cell types such as microglia (~10% of brain cells) may be significant mediators of ND pathogenesis, but are very difficult to detect and distinguish cell-type-specific changes using bulk proteome analysis. By conducting more focused (including cell-type or compartment-specific) proteomic analyses, we may overcome these barriers, and prove more detailed, targeted, and ultimately valuable insights into local changes during ND pathogenesis (Rangaraju et al., 2018; Rayaprolu et al., 2020).

### Cell-Type Specific Proteomics

The brain is composed of distinct cell types that react and contribute *via* specific mechanisms to pathogenic triggers. Understanding how a disease effects different cell types' proteomic landscapes could be very valuable to elucidating how cellular mechanisms contribute to disease and would eventually provide therapeutic insights. Bulk proteomic analysis cannot directly resolve cell-type specific changes because changes in cell-types which comprise the minority of brain cells gets diluted or under-sampled in bulk analysis. We thus suggest an emphasis on investigating the proteome of specific cell-type populations in the brain.

One reason for investigating a cell-type specific proteome is the implication of specific cell types in modules identified by bulk network proteomics. A large proportion of co-expression modules with associations with AD pathology identified by network-based approaches in AD pathogenesis were found to be enriched in cell-type specific markers (Higginbotham et al., 2020). Specifically, glia-enriched modules were increased in AD, and neuronal modules were decreased in AD. These cell-type assignments derive from a gene set enrichment analysis which is followed by assignment of known cell-type specific markers to the various modules (Eden et al., 2009; Zambon et al., 2012). One important module identified in several bulk network proteomic analyses is enriched with astrocytic and microglial proteins and highlights several “hub proteins” implicated in neuroprotection (Seyfried et al., 2017; Johnson et al., 2020; Swarup et al., 2020). This module is seen to increase in both asymptomatic and symptomatic AD, which suggests that these glial cells play a critical role in neuroimmunity against the progression of ND pathogenesis (Seyfried et al., 2017). The implication of specific cell types in modules of bulk proteomic analyses serves as a basis to investigate the network-based proteome of specific cell types as well as their correlation to disease progression.

In addition to the implication of specific cell-types by modules identified in the bulk proteomic analyses, there exists substantial evidence to suggest that disease pathogenesis both affects and is affected by different cell types in a cell-type specific manner. Here we will provide a brief description of the manner in which specific cell types are distinctly affected in neurodegeneration.

### Microglia

Microglia are phagocytic immune cells that comprise ~10% of adult CNS cells (Seyfried et al., 2017). The manner in which neuroinflammation contributes to and is affected by ND pathogenesis is partly microglia dependent, identifying microglia as a possible modulator of disease. Hickman et al. identify three primary roles of microglia: sensing their environment, conducting physiological housekeeping, and to protect against injurious agents (Hickman et al., 2018). Microglia recognize pathogen-associated molecular patterns (PAMPs) and damage-associated molecular patterns (DAMPs) and produce proinflammatory cytokines which help the CNS to recognize pathological agents and phagocytose them, while also recruiting other cells to participate. While these are neuroprotective mechanisms, pro-inflammatory cytokines activate resting microglia to produce pro-inflammatory factors which may be detrimental in NDs. This heterogenous microglial response produces microglia that have been broadly characterized as neurotoxic and neuroprotective (Kwon and Koh, 2020); although these simplistic characterizations do not capture the heterogeneity seen within microglia in human and non-human brain with or without pathology (Keren-Shaul et al., 2017; Masuda et al., 2020; Rayaprolu et al., 2020). The understanding of the contribution of microglia in ND pathogenesis is complicated by the presence of the complex and heterogeneous microglial phenotypes. Much remains unknown regarding the manner in which microglia, including homeostatic, pro-inflammatory and anti-inflammatory phenotypes or states, progress during ND pathogenesis.

### Astrocytes

Astrocytes are the most abundant glial cell-type in the CNS (Freeman and Rowitch, 2013). These cells are very dynamic and perform many different roles including maintenance of the blood brain barrier, formation and maintenance of neuronal synapses, and tissue repair after injury (Phatnani and Maniatis, 2015; Strooper et al., 2016). Additionally, astrocytes aid microglia in mediating the inflammatory response of the CNS (Vainchtein and Molofsky, 2020; Baxter et al., 2021). In reaction to pathophysiological changes in AD, astrocytes undergo “astrogliosis” which is accompanied by cellular proliferation and hypertrophy. Astrogliosis responds in stimuli-specific manners in different individuals, specific anatomical microdomains, and NDs (Zamanian et al., 2012; Phatnani et al., 2013; Liddelow and Barres, 2017; Jiwaji et al., 2022; Ziff et al., 2022). During astrogliosis, astrocytes release inflammatory modulators and various neurotropic factors. Similar to the aforementioned microgliosis, the factors released by astrocytes can be either neuroprotective or neurotoxic.

Astrocytes are also implicated in the pathogenesis of PD by genetic studies. Of the 17 monogenetic mutations found to be associated with PD by Herdandez et al., 8 are shown to have roles in astrocytes biology (Hernandez et al., 2016; Booth et al., 2017). Among the 8 protein products of these genes is DJ1 which is encoded by the *PARK7* gene (Hernandez et al., 2016). In astrocytes, the DJ1 protein regulates the assembly of lipid rafts which are membrane microdomains involved in endocytosis, exocytosis, and signal transduction (Simons and Ehehalt, 2002; Kim et al., 2013b, 2016). Mutations in the *PARK7* gene result in degradation of lipid raft proteins, and thus dysfunctional astrocytes which contribute to PD pathogenesis (Kim et al., 2016).

Astrocytes are further implicated in many tauopathies including Progressive Supranuclear Palsy, Corticobasal degeneration, and Aging-related tau astrogliopathy, by the presence of tau aggregates in diseased astrocytes (Togo and Dickson, 2002; Kouri et al., 2011; Kovacs et al., 2016; Reid et al., 2020). While Tau aggregates are typically found in neurons, their localization to astrocytes in these NDs implicate that in disease pathogenesis astrocytes internalize exogenous tau and may have roles in propagating this pathology.

### Oligodendrocytes

Oligodendrocytes generate and maintain the lipid-rich myelin sheaths which insulate neuronal axons and facilitate rapid signal transduction (Strooper et al., 2016; Kipp, 2020). Several studies have revealed myelination disfunction in the early stages of NDs which implicate oligodendrocytes in ND pathogenesis (Scheltens et al., 1992; Bartzokis, 2011; Tosto et al., 2015; Mot et al., 2018). Using magnetic resonance imaging (MRI)-based studies, scientists have shown that the myelin changes occurring with aging is exacerbated in NDs (Scheltens et al., 1992; Bartzokis, 2004; Tosto et al., 2015). Furthermore, other MRI studies have demonstrated that individuals with the *APOE4* allele, the strongest known genetic risk factor of AD, have increased myelin breakdown in AD (Bartzokis, 2011). Yet another recent study has demonstrated that white matter lesions attributable to myelination dysfunction have been found in individuals in the early stages of NDs (Tosto et al., 2015). In addition to the correlation of myelin breakdown with early ND pathogenesis, it has also been demonstrated that the reforming and maturation of myelin sheaths is significantly altered in the presence of amyloid-beta peptides, one of the two essential hallmarks of AD (Dean et al., 2017; Papuc and Rejdak, 2020). This implies that myelination is affected in the AD brain not only by myelin breakdown, but also in the inhibition of homeostatic myelin reformation.

### Neurons

Neurodegeneration is characterized by the progressive loss of function and eventual death of neurons and synapses in the nervous system. Thus, neurons are a natural focal point of cell-type specific studies related to NDs. Neuronal death occurs in a disease specific manner in many NDs (Gorman, 2008). In AD, neuronal death begins in the entorhinal cortex and hippocampus, and later is found in other areas of the cerebral cortex as the disease progresses (DeTure and Dickson, 2019). In contrast, PD is characterized by a loss of dopaminergic neurons focally in the substantia nigra (Kinoshita et al., 2015). It would be very interesting to compare neuron-specific proteomes with different disease-specific phenotypic traits to understand which characteristics make neurons vulnerable to specific diseases. Additionally, it would be important to compare the proteomes of neurons from different brain regions within specific phenotypes to identify mechanisms of regional vulnerability and resistance of neurons to different NDs.

In the sections above, we have demonstrated that neurodegeneration develops *via* cell-type specific mechanisms, although much remains unknown regarding the details of these mechanisms. We propose that by investigating the proteome of specific cell types using network-based approaches, we will obtain a much greater understanding of how cell-types mediate disease at different stages and thus, will understand the network of biological processes interacting across cell types at each stage of disease.

Furthermore, while we have discussed both that neurodegeneration is mediated by specific cell types and that several nuclear mechanisms are dysfunctional in neurodegeneration, we do not know what how these nuclear mechanisms functionally vary with cell type and disease state. We suggest that a proteomic analysis of cell-type specific nuclei, which has become possible with emerging technologies, will elucidate gaps in our understanding of both cell-type specific disease states and the how the nucleus contributes to neurodegeneration.

## Cell Type-Specific Nuclear Proteomics From Post-Mortem Human Brain

Analyses of human brain tissue at the proteomic level have been mostly performed at the bulk brain level rather than at the level of specific cell types. This is due to technical limitations related to isolating pure and intact cell populations from frozen brain tissue. While microglia and other cell types can be isolated intact from post-mortem brain, neurons do not survive cell isolation approaches, and further, the immense cellular and tissue complexity of human brain as compared to mouse brain, poses additional technical challenges (Bohlen et al., 2019). Mouse models have often been used, because of the great potential for experimental manipulation of rodent models and control over tissue quality. While mouse models have provided invaluable information into ND pathogenesis, there are limitations due to substantial species differences, which exist at molecular, cellular and pathological levels (Maloney et al., 2007; Monaco et al., 2015; Breschi et al., 2017).

Recent advances in single cell nuclear transcriptomics from mouse and human brain have revealed cellular heterogeneity in healthy, aging and disease states (LoVerso and Cui, 2016; Mathys et al., 2019; Lau et al., 2020; Cid et al., 2021), but protein-level inferences cannot be drawn confidently from mRNA levels. Since intact cells cannot be isolated from frozen or fixed human brain, cellular proteomic insights into human ND from human post-mortem tissues are very limited. However, the nucleus of the cell retains its structural integrity even after frozen tissue is thawed. This unique property allows the isolation of intact nuclei from frozen brain tissue for downstream analyses.

**Figure 3:**
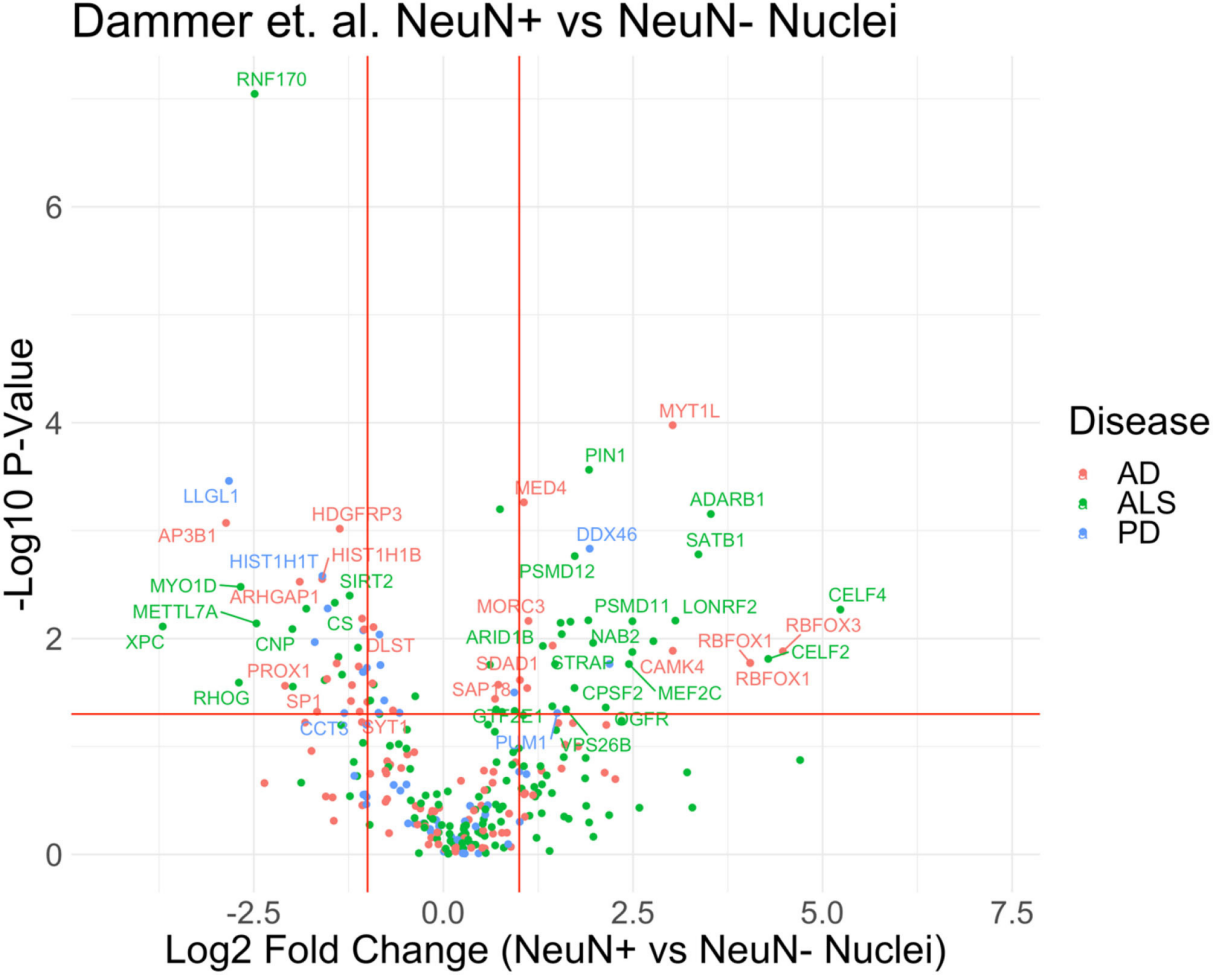
Proteomics of neuronal and non-neuronal nuclei from frozen human post-mortem brain. Volcano plot of proteomic differences between NeuN^+^ vs. non-NeuN Nuclei with highlighted disease markers that have *p*-value <0.05 and fold change >2. The reannotated data demonstrates that proteins found in cell-type specific nuclear populations are correlated to disease. List of MAGMA genes associated with each ND displayed in this figure can be found in Supplementary Table 1.

In a study by Dammer et al., NeuN-positive (neuronal) and NeuN-negative (non-neuronal) nuclei were isolated from frozen post-mortem human brain by wide-clearance dounce homogenization, centrifugation, and then flow cytometry based sorting of nuclei, followed by mass spectrometry proteomic analyses. Using this approach, termed Fluorescence Activated Nuclear Sorting (FANS), Dammer et al. identified 487 nuclear proteins that were highly enriched in NeuN+ve nuclei and 420 which were highly enriched in NeuN-ve nuclei. Using these differentially expressed nuclear proteins, we cross referenced these lists against known genetic risk factors for AD, PD and ALS (Figure 3, Supplementary Data 1) (Nalls et al., 2014; Zhang et al., 2014; Sharma et al., 2015; Iacoangeli et al., 2020; Sarkar et al., 2020), and identified several disease-relevant markers expressed in nuclei of neurons and non-neuronal cells that could serve as nuclear and cell type-specific markers for future FANS approaches. If these markers can be leveraged for further optimization and coverage of brain cell types, the preservation of nuclear proteomic integrity in the frozen human brain can be leveraged to investigate nuclear specific proteomic mechanisms of NDs with cell type-specific resolution. Accordingly, a recent protocol describes a method to extend FANS to non-neuronal cells using markers such as SOX10 (oligodendrocyte), IRF8 (microglia) and the absence of these markers to purify nuclei from frozen human brain in a cell type-specific manner (Policicchio et al., 2020).

## Proposed Method for Cell Type-Specific Nuclear Proteomics

The study of the nuclear proteome offers intriguing possibilities, not only because of its enabling of cell-type specific proteomics, but additionally because of the ability to investigate nuclear alterations that occur during ND pathogenesis that may be missed by whole cell or bulk tissue proteomics. Alterations in protein can be linked to additional omic data obtained from additional samples of the same purified nuclei. As previously mentioned, numerous studies implicate nuclear pathways in NDs, including but not limited to: NCT, chromosomal stability, RBPs, and nuclear inclusion bodies. Thus, investigating cell-type specific nuclear proteomes would provide us with a better understanding of how these biological pathways, and specific cell phenotypes are altered in a cell type specific manner over the course of NDs.

Recently, Nott and colleagues designed a protocol aimed at isolating cell type specific nuclei for the purpose of genomic and epigenomic investigation. The authors of this new protocol asserted that nuclei isolated using their protocol can be used to identify chromatin features including chromatin architecture and to identify the binding sites of transcription factors using chromatin immunoprecipitation-sequencing (ChIP-seq) (Nott et al., 2021). While the protocol proposed by Nott et al. aimed to isolate the transcriptome of cell-type specific nuclei, a study conducted by Dammer et al. used similar methods to isolate the proteome of neuronal specific and non-neuronal specific nuclei. This proteomic study implemented LC-MS/MS which identified 1,755 proteins, ~20% of which were significantly up or down regulated in the neuronal cell populations compared to the non-neuronal cell populations (Dammer et al., 2013). The protocol proposed by Nott et al. attempts to label and purify nuclei of microglia, astrocytes, and oligodendrocytes in addition to neurons. Specifically, they used the nuclear markers NeuN for neurons, PU.1 for microglia, and Olig2 for Oligodendrocytes (Nott et al., 2021).

**Figure 4:**
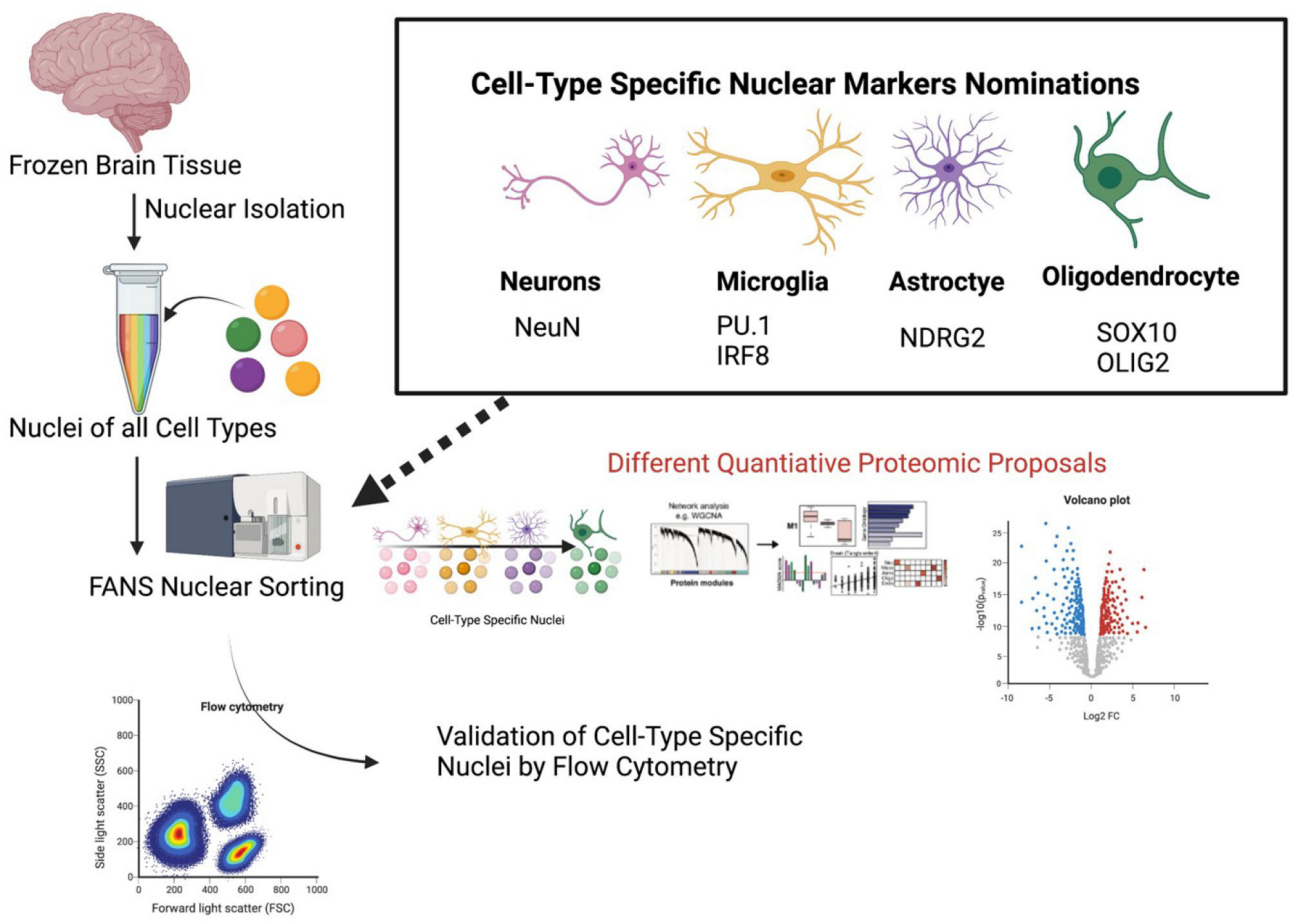
A proposed pipeline for cell type-specific nuclear proteomics of human brain. Proposed specific cell-type specific nuclear markers for flow cytometry are listed. A proposed workflow for nuclear proteomics is also outlined once nuclei have been purified.

While these experiments aimed to investigate the nature of cell-type specific alterations at different levels, they both implemented a similar workflow to isolate the cell-type specific nuclei. Both studies prepared the tissue, isolated pure nuclei, and then implemented antibody tagging of the nuclei to distinguish between cell-types, followed by fluorescence activated nuclei sorting (FANS) to sort the nuclei. Dammer et al. found that FANS not only allowed cell-type specific nuclear sorting but additionally reduced non-nuclear protein contamination (Dammer et al., 2013) when comparing to the proteome of unsorted nuclei. Based on these studies, we propose a workflow for CNS cell type-specific FANS (Figure 4) to decipher cell type-specific nuclear mechanisms of NDs using frozen human post-mortem brain samples.

The proposed FANS approach takes advantage of the integrity of the nucleus in the brain, even from archived frozen samples. This is important because cellular architecture is typically lost during thawing frozen brain tissue, which limits the ability perform proteomic analyses of intact cells, or to subject them to down-stream cellular purification using cell surface markers. This is in stark contrast to handling fresh brain samples which are amenable to cell type dissociation and intact cell isolation for bulk and single cell omics approaches. Rapidly evolving mass spectrometry-based proteomics strategies (eg. proteoCHIP and single cell proteomics by mass spectrometry or SCOPE-MS) are also promising approaches that may be capable of obtaining deep proteomes from single cells or nuclei or pools of small numbers of nuclei (10–100) from brain (Budnik et al., 2018; Zhu et al., 2018). Apart from mass spectrometry, high-dimensional proteomic profiling methods, such as mass cytometry by time-of-flight, and high-dimensional flow cytometry can also be used to capture several parameters from single cells or single nuclei (Korin et al., 2018; Weber et al., 2019). From frozen human brain tissues, it is possible that future single nuclear proteomics approaches can complement or serve as an alternative approach to FANS. As with current single nuclear transcriptomics approaches, the depth of the proteome from single nuclear omics is likely to be an order of magnitude lower than depth of a bulk nuclear proteome. A limitation of the FANS approach is that it is biased against cytosolic proteins by design, and will therefore not capture cellular mechanisms that occur outside the nucleus. However, when comparing nuclear proteomes of distinct cell types from disease cases and controls, relative abundance or absence of nuclear proteins will provide clues toward protein trafficking and mislocalization, which will warrant additional studies to verify these findings.

## Summary

We have reviewed the importance of key nucleus-specific mechanisms and of individual cellular contributions in the pathogenesis of NDs. Despite advances in bulk and cell type-specific transcriptomics and bulk brain proteomics of human NDs, there is an immense knowledge gap in cellular mechanisms of NDs occurring at the proteomic level. Deciphering these cell type-specific proteomic mechanisms in human NDs can provide a deeper understanding of disease mechanisms and identify therapeutic targets for NDs. While cell type-specific proteomics of intact cells from human post-mortem brain is technically challenging, the nucleus of the cell is preserved even in archived human brain samples, representing an untapped resource to investigate molecular mechanisms of NDs specifically occurring within the nucleus. We have highlighted the FANS approach as a promising method for nuclear proteomics of neurons and non-neuronal cells from post-mortem human brain. While further studies are warranted to improve this method, the cell type-specific FANS approach holds great promise for proteomic analyses of diverse CNS cell types in human neurodegeneration.

## Author Contributions

RN and SR conceptualized, drafted, and edited the manuscript. ED, JS, and NS critically reviewed and edited the manuscript. All authors contributed to the article and approved the submitted version.

## Funding

Research reported in this publication was partly supported by the National Institute on Aging of the National Institutes of Health and National Institutes of Health: R01 NS114130 (SR), R01 AG075820 (SR, NS), and R01AG061800 (NS).

## Conflict of Interest

The authors declare that the research was conducted in the absence of any commercial or financial relationships that could be construed as a potential conflict of interest.

## Publisher's Note

All claims expressed in this article are solely those of the authors and do not necessarily represent those of their affiliated organizations, or those of the publisher, the editors and the reviewers. Any product that may be evaluated in this article, or claim that may be made by its manufacturer, is not guaranteed or endorsed by the publisher.

## Abbreviations

ND: Neurodegenerative disease
AD: Alzheimer's disease
ALS: Amyotrophic lateral sclerosis
PD: Parkinson's disease
FTD: Frontotemporal dementia
CNS: Central nervous system
FACS: Fluorescence-associated cell sorting
MS: Mass spectrometry
NCT: Nucleocytoplasmic transport
CIN: Chromosomal instability
GWAS: Genome-wide association study
RNA: Ribonucleic acid
RBP: RNA binding protein
LATE: Limbic-predominant Age-related TDP-43 Encephalopathy
hnRNPs: heterogeneous nuclear ribonucleoproteins
SR: Serine/Arginine-Rich
NFT: Neurofibrillary tangle
SCOPE-MS: Single cell proteomics by mass spectrometry.
